# Oral Hygiene Practices, Knowledge, and Self-Reported Dental and Gingival Problems with Rural-Urban Disparities among Primary School children in Lilongwe, Malawi

**DOI:** 10.1155/2021/8866554

**Published:** 2021-03-09

**Authors:** F. Mlenga, E. G. Mumghamba

**Affiliations:** ^1^Department of Orthodontics, Pedodontics and Community Dentistry, Muhimbili University of Health and Allied Sciences (MUHAS), P.O. Box 65014, Dar-es-Salaam, Tanzania; ^2^Department of Restorative Dentistry, School of Dentistry, Muhimbili University of Health and Allied Sciences (MUHAS), P.O. Box 65014, Dar-es-Salaam, Tanzania

## Abstract

**Aim:**

To determine oral hygiene practices, knowledge, and experience of dental caries and gingival problems among urban and rural primary schoolchildren in Lilongwe District, Malawi. *Methodology*. This was an analytical and quantitative descriptive cross-sectional study. Four urban and four rural primary schools were conveniently sampled. Pupils aged 11–14 years (*n* = 409) were recruited using self-administered structured questionnaire. Data were analyzed using SPSS program v20.0.

**Results:**

Out of 409 pupils, most of them had knowledge that dental caries is caused by consumption of sugary foodstuffs (91.4%), toothache is a symptom of dental caries (77.6%), gingivitis is caused by ineffective tooth brushing (92.7%), and gingival bleeding is a sign of gingivitis (85.3%). Most pupils experienced toothache (30.8%); many of them had parents with secondary education and above (35.0%) compared to those with primary education (23.5%). 24.4% experienced gingival bleeding with higher percentages from urban (30.1%) than rural (18.5%) schools. Plastic toothbrush users (95%) overshadowed chewing stick users (24.9%). The use of chewing stick was significantly higher in rural (49%) than in urban (1.9%) schools. Likewise, tooth brushing before bed was significantly higher in rural (33%) than in urban (17.2%) pupils. The use of toothpaste during tooth brushing was significantly higher among urban (91.9%) than among rural (64%) pupils. The prevalence of tongue cleaning was 70.2%, and the differences were significantly higher among pupils who had parents with secondary and higher education in urban schools and among pupils aged 11–12 years in comparison with their counterparts.

**Conclusions:**

Most pupils reported cleaning their teeth regularly, mostly using plastic toothbrush rather than chewing stick, using toothpaste, and having adequate knowledge about dental caries and gingival disease, and a quarter of them had suffered from these diseases with evidence of rural-urban disparities. Integration of oral health in school health promotion program and further research on its impact on oral health status are recommended.

## 1. Introduction

Dental caries and periodontal disease are among the commonest diseases that affect the oral cavity, hence becoming one of the major public health problems that cause significant social impact [1]. Periodontal diseases always progress from an established gingival lesion, gingivitis [2, 3]. However, not all gingival lesions do progress to periodontal diseases [4].

It is estimated that about 60–90% of children worldwide suffer from dental caries leading to pain and discomfort [5]. The negative impact and burden of oral diseases restrict activities in schools and home, leading to the loss of many potential working hours and more than 51 million school hours worldwide [6, 7], respectively. Oral hygiene is the practice of maintaining the mouth clean and healthy so that oral diseases affecting the oral cavity and its surrounding structures are prevented. Maintaining good oral hygiene is considered to be a lifelong habit, and these oral habits are said to begin in an early stage of life [6]. According to Owino and coworkers [8], oral hygiene plays an important role in the prevention of dental caries and periodontal diseases.

Dental caries is a disease of hard tissues of the oral cavity characterized by episodes of demineralization and remineralization over a period of time [9]. Tooth decay (dental caries) is a multifactorial disease, caused by the interaction between the tooth surface, the bacterial biofilm (dental plaque), and the presence of sugars from food [10]. If dental caries is left untreated, decay can lead to extensive destruction of the tooth. Signs and symptoms of dental caries include toothache and dental abscess formation that may lead to septicemia [10, 11]. Action to protect the tooth surface can be taken by ensuring adequate exposure to fluoride, for example, by using fluoride toothpaste, or fluoridating water supplies, and also good oral hygiene practices [10]. Decrease of dental caries prevalence among children and adolescents has also been reported to be a result of health promotion programs through schools as recommended by the World Health Organization [12].

Gingival disease that is completely reversible for the majority of patients is the commonest disease in children and adolescents and is characterized by the presence of gingival inflammation without detectable bone loss or clinical attachment loss of the periodontal tissue at the junctional epithelium [10, 13]. The most important local predisposing factor in children is poor oral hygiene which stems from children's dependence on adults' assistance with routine oral hygiene measures. The situation can be controlled with regular dental visits where oral health education is reinforced [13]. Many of these oral diseases are preventable when they are recognized early. However, there is limited knowledge of the causes and prevention of oral disease in several countries in children, their parents, and teachers [14].

In Malawi, there is limited information on the status of oral health problems. However, one retrievable study done in 2016 indicated that the prevalence of dental caries among 12-year-old children was 19.1% and that of gingival bleeding was 13% in the same age [15]. Following this scarcity of information on oral health, there is a need to conduct a study to assess oral hygiene practices, knowledge, and experience of dental caries and gingival disease among primary schoolchildren in Malawi.

According to the 2018 Census report, Malawi that is located in southern Africa and shares its borders with Mozambique, Zambia, and Tanzania has a total population of 17,563,749 people whereby urban residence was 16% and rural residence 84% [16]. Across the whole country of Malawi, dental services are delivered in four main referral central hospitals (Mzuzu, Kamuzu-Lilongwe, Zomba, and Queens Elizabeth-Blantyre), 28 district hospitals, and 31 health centers (20 in urban and 11 in rural areas). Dental personnel include the dentists (more than 90% work in referral hospitals and medical institutions in urban and less than 10% in district hospitals). Most of the dental therapists work in the district hospitals. For the rural health centers, even though they do not have dental equipment like dental chairs, through outreaches they are visited routinely on a monthly basis by dental personnel to render emergency dental care especially tooth extraction. Sectors that provide dental services in Malawi are the Government (Public), private for profit (PFP), and private not for profit (PNFP) faith and nonfaith organizations as well as individuals [17].

## 2. Materials and Methods

### 2.1. Study Design, Place of Study, and Participants

This was an analytical and quantitative descriptive cross-sectional study. This study was done in Malawi, a country situated in the African continent and sub-Saharan region. The target population was the primary school-going children areas in Lilongwe district. The sample size was 409 as calculated using the following formula: *N* = *Z*^2^*P* (1−*P*)/*D*^2^ , where *N* is the minimum sample size required; *Z* is the standard normal deviation of the significant level (taken to be 1.96); *P* is the estimated proportion of school-going children in the previous study population which in this study will be 39.9% according to Okemwa et al. (2010) study; *D*^2^ is the maximum likely error in the study, set at 0.05. Thus, *N* = (1.96)^2^ x 0.399 (1–0.399)/0.05^2^ (*N* = 368 participants). To adjust for nonresponses, 10% of the calculated sample size was added to the N as follows: formula: *n* = *N* (100%)/100%–10%; *n* = 368 (100)/100–10% (*N* = 409 participants).

Eight easily accessible primary schools were conveniently sampled including four urban and four rural settings. The specific primary schools from the urban areas were Chimutu, Kabwabwa, Chilinde, and Mlodza while the rural schools included Mitundu, Chiwili, Tsabango-2, and Gumbi. The maximum number of “50” was used as the limit of number of pupils to be examined from each of the eight schools with the goal of attaining at least 400 total study participants. Only pupils aged 11–14 years of standard/class 6–8 that consented were recruited for the study. The pupils were then selected by random sampling method whereby some small pieces of paper were written with some numbers from 1 to 50 and other pieces of paper were blank as they did not have any number. All who picked the papers with the numbers 1–50 were given the questionnaire to answer. All pupils that were sick and absent from school on the day of administering the data research questionnaire were excluded from the study. The study period was from September and October 2016.

### 2.2. Data Collection Tool and Data Management

A self-administered structured questionnaire was developed in English and afterwards translated into “Chichewa”, the local language of the study participants in Lilongwe, Malawi. Using MUHAS experience, the questionnaire was broadly divided into five parts: (1) sociodemographic data consisting of age, sex, and educational level of their parents, (2) oral hygiene practices with 6 questions, (3) knowledge of dental caries causes with 8 questions and prevention of dental caries with 3 questions, (4) knowledge of gum disease causes and prevention each with 2 questions, and (5) experience with 4 questions. Knowledge score above 50% was considered as adequate knowledge among pupils and below this limit was taken as poor knowledge. Due to constraints in resources, piloting and validation of the questionnaire were not done.

The principal investigator (FM) visited the selected schools and distributed the self-administered structured questionnaires to the randomly selected pupils in a classroom setting, accordingly. The collected data was entered into a computer, and data cleaning process was undertaken systematically. The Statistical Package for Social Sciences (SPSS) version 20.0 was employed to analyze the data. Frequency and cross-tabulation tables were generated. Data transformation was done in particular for dichotomization of variables that had more than two options, for example, pupil's age (11–12 years versus 13–14 years), class of the pupils (upper class versus lower class), residence (urban versus rural), and level of education of the pupils' parents (secondary education and above versus primary education and below). Chi-square (*X*^2^) test or Fisher's exact test was used to detect significant differences between the categorical groups, but, for continuous data, Student's *t*-test was used, accordingly.

For binary logistic regression analyses, we used the dichotomized variables, and the coding was that zero (0) was assigned to an advantageous, normal, or healthy situation and a code of one (1) was given to any disadvantageous, abnormal, disease situation. For example, any subject with a sign for a disease or condition under study was given a code number of “one (1)”, whereas a study subject without any sign of that disease or condition was given a code number of “zero (0)”. All those variables that were dichotomized for the binary logistic regression analyses were subjected to descriptive statistics in particular cross-tabulation. A selection of variables to be entered to the logistic regression analysis “model” was those that in the cross-tabulation had a significant *P* value; that is, *P* < 0.05. In addition, all variables that were thought to be important as predisposing factors for the dependent variables (gingival bleeding and “toothache”) were also entered in the model. The backward stepwise (Wald) logistic regression method was chosen for analysis. The final iterations of the backward stepwise (Wald) model were reported here as final results. In all the analyses, the statistical significance level was set at “*P* < 0.05”.

## 3. Results

### 3.1. Study Participants

The total number of study participants was 409 primary school pupils. There were approximately equal proportions of pupils from urban (51.1%) and rural (48.9%) schools but with slightly more females (52.8%) than males (47.2%, Table 1). The age structure of the pupils ranged from 11 to 14 years (mean age: 12.75 ± 1.15 years). The mean age was slightly higher among the rural (13.12 ± 1.04 years) than the urban pupils (12.40 ± 1.14 years) (*T*-test, *P* < 0.001). There was no significant difference of age in years among the males (12.84 ± 1.17 years) as compared to females (12.68 ± 1.12 years).

### 3.2. Oral Hygiene Practices

Oral hygiene practices among the participants are shown in Table 2. Among the 409 studied primary school pupils, the majority cleaned their teeth by brushing (91.4%), but others used finger (9.8%) and some just rinsed with water (10.3%). The frequency of tooth brushing was twice/day (27.9%) or thrice/day (48.7%). Collectively, 76.6% of the pupils practiced tooth brushing two times per day. Toothbrush use was significantly higher among pupils in urban (95%) than rural schools (*P* < 0.001). Tooth brushing was mostly performed before breakfast (91.9%), and brushing before going to bed was practiced by only about a quarter of all pupils (24.9%, Table 2). There was significantly higher proportion of pupils from rural (33%) than urban (7.7%) schools who brushed their teeth before going to bed (*P* < 0.001).

The majority of the pupils (91.9%) used plastic toothbrush, and only about a quarter used chewing sticks (24.9%). The proportion of chewing stick users was significantly higher among pupils in rural (49.0%) than in urban (1.9%) schools (*P* < 0.001) and also among pupils who had parents with primary (46.3%) education compared to those with secondary or higher (12.7%) education (*P* < 0.001).

Agents used during tooth brushing included toothpaste (78.2%), ashes (21%), and salt (16.4%). Toothpaste use was significantly higher among pupils in urban (91.9%) than in rural (64.0%) schools (*P* < 0.001). Tongue cleaning was practiced by most of the pupils (70.2%) and was significantly higher among pupils in urban (84.7%) than rural (55.0%, *P* < 0.001) schools, among those from parents that had secondary or higher (78.8%) than primary education (55.5%, *P* < 0.000), and among those in the 11–12 years (77.9%) than the 13–14 years (64.6%) age group (*P* < 0.01, Table 2). There was no statistically significant difference between males and females as regards to various aspects of oral hygiene practices (Table 2).

### 3.3. Knowledge about Causes, Symptoms, and Prevention of Dental Caries and Gingival Disease

The distribution of study participants in relation to their knowledge about causes, symptoms, and prevention of dental caries and gingival disease is shown in Table 3. Among the 409 study participants in the study, the majority had knowledge about causes of dental caries (92.1%), symptoms of dental caries (72.4%), and prevention of dental caries (85.6%). The study participants that had knowledge about causes of gingivitis were 379 (92.7%), about symptoms of gingivitis were 85.4%, and about prevention of gingivitis were 92%. Pupils with knowledge about symptoms of dental caries were more in rural (81.0%) than in urban (64.1%) schools (*P* < 0.001). The proportion of pupils that had knowledge about prevention of dental caries was slightly higher in urban (89%) than in rural (82%) schools (*P* < 0.05), and likewise, for causes of dental caries, the proportion was slightly higher among pupils from parents that had education at secondary school and above (95%) compared to primary level and below (87.2%) (*P* < 0.001). The proportion of pupils that had knowledge about prevention of gingivitis was slightly higher in urban (95.2%) as compared to rural (88.5%) schools (*P* < 0.05) There was no sex difference in the knowledge about causes, symptoms, and prevention of both dental caries and gingival disease.

### 3.4. Toothache Experience, Gingival Bleeding, Gingival Swelling, and Disturbances in Class

The distribution of study participants in relation to problems they had experienced especially toothache, gingival bleeding, gingival swelling, and disturbance in class attendance for the past 6 months is shown in Table 4. Among the 409 study participants, oral health problems experienced for the past 6 months were toothache (30.8%), gingival bleeding (24.7%), gingival swelling (21.7%), and disturbances in class (18.7%). The problem of toothache was experienced more by pupils in the 11–12 years (37.2%) than the 13–14 years age group (26.2%, *P* < 0.05). Pupils from parents that had secondary education and above reported more toothache problems (35.0%) than those from parents with primary education and below (23.5%; *P* < 0.05). More pupils from urban (40.2%) than rural (21.0%) schools reported to have suffered from toothache (*P* < 0.001). Gingival bleeding as well as gingival swelling was reported by more pupils in urban (30.1% and 27.3%) than rural (18.5% and 16.0%) schools (*P* < 0.05 and *P* < 0.01, respectively). There were no gender differences as regards to toothache, gingival bleeding, and disturbance in class in this study population although there was a slightly higher percentage of females (31.5%) than males (27.5%) who suffered from toothache, and more females (20.8%) experienced much disturbance in class due to toothache problem than males (16.6%) (Table 4).

The binary logistic regression analyses (backward stepwise Wald) final model for toothache and gingival bleeding in relation to oral hygiene knowledge, oral hygiene practices, and demographic factors among primary schoolchildren is shown in Table 5. The most significant factors associated with toothache were urban residence (odds ratio (OR): 2.806, 95% confidence interval (CI): 1.765–4.461, *P* < 0.001) and “not using plastic toothbrush” (OR: 2.624, 95% confidence interval (CI): 1.194–5.767, *P* < 0.05). As regards to gingival bleeding, the most significant factors associated with it were “not using” plastic toothbrush (OR: 3.395, 95% CI: 1.540–7.454, *P* < 0.01), “not brushing” before breakfast (OR: 2.165, 95% CI: 1.012–4.632, *P* < 0.05), and urban residence (OR: 2.192, 95% CI: 1.333–3.600, *P* < 0.01).

### 3.5. Source of Information about Tooth Decay and Gingival Disease

Sources of oral health education in relation to dental caries and gingival disease that had an impact on the studied schoolchildren in general were the dentist (57.2%) and parents (45.5%) followed by teachers (36.2%) and a PNFP organization “Teethsavers International” (31.1%). The least acknowledged source of information was the radios (22.2%) and newspapers (9.3%). Dentist's role in giving oral health information was almost equally recognized in both urban (58%) and rural (56%) areas as well as among males (57.5%) and females (56.9%). The appreciation of parents and Teethsavers International (TSI) as sources of information was higher in urban (51.7% and 42.1%) than rural areas (39% and 19.5%, *P* < 0.05 and *P* < 0.001, respectively).

### 3.6. Rural-Urban Disparities

There were significantly more pupils in rural than urban schools that used rinsing with water, finger, and chewing stick for tooth cleaning (Table 2). Also rural as compared to urban schools had higher proportion of children that brushed their teeth at meal-time and before going to bed, using salt, ash, and sand (Table 2) and that had knowledge about symptoms of dental caries (Table 3). On the other hand, the proportion of pupils that received oral health information through TSI was low in rural compared to urban schools.

Urban compared to rural schools had significantly higher proportion of pupils that performed tooth brushing practice and used plastic toothbrush and toothpaste as well as tongue cleaning (Table 2). Also urban schools had higher percentages of pupils that had knowledge of prevention of dental caries and gingival disease (Table 3) and experienced toothache, gingival swelling, and gingival bleeding (Tables 4 and 5).

## 4. Discussion

This report was based on a descriptive cross-sectional study among pupils aged 11–14 years from urban and rural primary schools in Lilongwe district, Malawi. The schools were conveniently sampled, for easy accessibility during data collecting with the consideration of constraints in funds and time to conduct the study. However, the studied pupils were randomly selected after stratification of rural and urban schools. The included urban schools were close to the city where parents of the studied pupils were of middle and high socioeconomic status and thus had the ability to own televisions and radios.

The proportion of rural schoolchildren in the current study that had knowledge about causes of dental caries was comparable to what was reported in Zambia [18] but slightly higher than those reported in Burkina Fasso [19] and in Kenya [20]. The high proportion of children in rural schools that had knowledge about causes of dental caries might have been contributed by “Teethsavers International” that provides oral health education in Malawi. Also pupils that had parents whose level of education was secondary and above and had good knowledge about causes of dental caries and prevention had higher knowledge than those who had parents with primary education or lower, and this can be attributed to the role of parents in educating their children.

Regarding knowledge about caries prevention, the current findings showed that most pupils, who were coming from urban schools and had parents that attained secondary level of education and above, had good knowledge compared to their counterparts. These results were similar to the study done in Zambia [18], India [21], and southwestern Michigan [22] where most of the children knew that fluoride toothpaste helps to protect teeth from decay, but were higher than those reported in Western China [23] and Nepal [24]. Good knowledge about caries prevention among pupils in the current study might be explained by the impact of teachers and TSI. The majority of the pupils who were in the upper classes and coming from urban schools had higher knowledge about prevention of dental caries compared to their counterparts in rural schools and being in the lower class. These findings are comparable to those reported in the neighboring country, Zimbabwe [25], and the differences might be accounted for by socioeconomic status as well as teachings in these classes.

Toothache is a symptom of dental caries. Most of the pupils aged 13–14 years, compared to their counterparts aged 11–12 years, had the knowledge that toothache is a symptom of dental caries, and the proportion was slightly lower than that reported in Zambia [18]. On the same issue, more pupils from rural schools who had parents with primary education and below, compared to their respective counterparts, reported to be aware that toothache is a symptom of dental caries, and similar findings were reported in Qatar [26]. The reason for this statistically significant difference is not known, but speculation includes the possibility that these pupils had the information from their parents and teachers as it is thought that in rural areas these people are closer to their children than it is the case in urban areas.

Compared to their counterparts, most pupils whose parents' level of education was secondary and above and who were coming from urban primary schools reported that they had experienced toothache. The possible explanation for this might be the fact that higher socioeconomic status is linked to higher purchasing power, thus having more chance to afford sugary foodstuffs. The association of urban residence with high toothache experience can be attributed to the availability of sugary foodstuffs that cause caries. These findings, in particular the toothache experience, were comparable to those reported in Western China [23], but were higher than what was found in Nigerian children [27].

The majority of the pupils in this study had the understanding that ineffective tooth brushing could cause gingival disease, and similar findings were reported in Zambia [18]. Also in our present study, there was a high proportion of pupils that had knowledge of prevention of gingival disease. Oral health education given to schoolchildren by TSI in Malawi, teachings received from the teachers as well as television and radio and school health programs might be among the contributing factors to the high awareness of causation and prevention of oral diseases.

Gingival bleeding on tooth brushing that signifies the presence of gingival disease was more prevalent among schoolchildren from urban than rural schools. The possible explanation for this might be that parents' care and time are limited in urban schools due to other more demanding activities that are characteristic of urbanization, thus undermining supervisory role to good oral hygiene practices that are conducive to good oral health. In addition to this, there is a challenge on diagnostic procedure of “how someone becomes aware of gum bleeding on tooth brushing”. The process means that on brushing teeth one has to spit the saliva on a certain surface in order to check for bloodstains. This approach might be easier in urban areas as it is assumed that, after brushing, children were spitting on white washing water sinks while, in rural areas, children were most likely to spit on the floor, where color contrast to “bloodstain” is much more difficult to comprehend. The findings from the current study on the magnitude of gingival bleeding on tooth brushing were slightly higher than those reported in Qatar [26]. Essentially, this shows that gingival bleeding on tooth brushing is a worldwide problem among other child populations. Also pupils from urban schools and whose parents had secondary level of education and above had experienced more gingival swelling compared to those from rural schools and whose parents' level of education was primary and below. Possible explanation for these findings one needs to understand among other things is that the environment and features surrounding the study area affect the pupils, meaning that pupils from urban areas were exposed to good oral health facilities with good pictorial presentations of gingival disease, electric light source, and mirrors that enable easier self-inspection of oral tissues than it is the case in rural schools.

The current study found that most of the pupils had good oral hygiene practices. The use of plastic toothbrush was higher among pupils in urban than rural schools. The findings were in agreement with those reported in Nepal [24], Bangladesh [28], India [29], Ambala, Haryana [30], Saudi Arabia [31], and Cameroon [32]. The possible explanation for these findings is that plastic toothbrushes are readily available in urban areas and that parents to these pupils had higher purchasing power compared to those coming from rural areas. Advertisements and other educational programs on television might have helped also more children in urban areas to gain knowledge about the use of toothbrush compared to those pupils in rural schools.

The current study indicated that most of the pupils were using toothpaste when brushing their teeth, and these findings were comparable to those from other child populations in India [33], Bangladesh [34], and Nigeria [35] but different to what was found in Kenya [20], where a small proportion of the pupils reported using toothpaste. In the current study, fewer children used ashes when brushing their teeth, and the findings differed from those reported in Bangladesh [34]. Less than one-third of the pupils in the current study were brushing their teeth twice per day, and this finding was comparable to what was found in Kenya [20] but different to what was observed in Qatar [25].

Most of the pupils in urban schools and pupils whose parents had secondary education and above were using plastic toothbrush and toothpaste and brushing their tongue compared to their counterparts. The socioeconomic status of parents is thought to be a contributing factor in this kind of distribution. Similar findings on oral hygiene practices were reported from Libya [36], where the majority of the children had parents with higher level of education.

Most of the pupils in the current study brushed their teeth before breakfast, few before bedtime and twice a day. These findings are similar to those reported in Asian countries [34, 37]. However, in our study, there were significantly more pupils in rural than urban schools that brushed before bedtime. The possibility that parents' control over their children was more effective in rural than urban schools might be the reason among others for this observation. Also, it was speculated that most of the urban pupils possibly spent more of their time watching television and hence had limited time to take care of their teeth particularly before bedtime. The practice of using chewing stick for tooth brushing was significantly higher in rural than urban pupils, and such findings were similar to what was found in other child population in the neighboring country [20].

The source of information about oral health education in relation to dental caries and gingival disease in the current study was largely and significantly from parents followed by TSI in urban compared to rural areas, and this can be explained by the fact that urban parents had attained secondary school education and higher and that TSI delivered its activities starting from the urban schools and slowly rolled to the rural areas.

Findings from our present study revealed a “rural-urban disparity” in oral hygiene practices, knowledge about causes and preventive measures of dental and gingival disease, and lastly a history of suffering from these diseases. The rural-urban disparity had earlier been reported to be a common observation in many African and Middle-East countries and needed to be ameliorated to benefit equally all social and economic strata [38].

These research findings originated from a cross-sectional study that employed a conveniently selected sample size due to constraints in resources and therefore had to be interpreted cautiously with the understanding of some inherent limitations. Firstly, the research tool to collect data was a self-structured questionnaire and therefore relied on answers given by the respondents. Measurement errors due to misinterpretation of some questions were subject to occur [25]; however to overcome this problem, the questions were worded in simple terms and translated to local language (Chichewa). Secondly, due to constraints in resources, the selected primary schools, both urban and rural, were picked up with some consideration of convenience for easy accessibility. Hence, the findings from this study cannot be generalized for the whole school going child population in Lilongwe district and the entire country.

## 5. Conclusions

The findings from the current study population showed that most of the pupils had adequate knowledge about causes and prevention of dental caries and gingival disease. The majority of the pupils self-reported to clean their teeth with fluoridated toothpaste using mostly plastic toothbrush rather than chewing stick, and about a quarter had suffered mostly from dental caries followed by gingival disease characterized by rural-urban disparities.

## 6. Recommendations

The current findings may be utilized as baseline information to design integrated school oral health and health promotion programs for primary school-going children and at the same time taking care of the rural-urban disparities. Further research using a representative sample on the impact of oral health knowledge and practices on oral health status is recommended. Schoolchildren in urban schools and those belonging to parents with secondary level education and higher should be prioritized as a population at high risk for dental caries and gingival disease when planning for oral health care services and preventive measures.

## Figures and Tables

**Table 1:** Distribution of study participants by sociodemographic characteristics.

Sociodemographic characteristics	Frequency (*n*)	Percentages (%)
Residence		
Urban	209	51.1
Rural	200	48.9

Sex		
Male	193	47.2
Female	216	52.8

Age group		
11–12 years	172	42.1
13–14 years	237	57.9

Standard/class		
Lower class	127	31.1
Upper class	282	68.9

Parents highest level of education		
Primary and below	149	36.4
Secondary and above	260	63.6

**Table 2:** Oral hygiene practices among the primary schoolchildren by sociodemographic factors (in percentages).

Oral hygiene practices	All respondents (*n* = 409)	Gender		Residence		Age group (years)		Parents' highest level of education	
		Male (*n* = 193)	Female (*n* = 216)	Urban (*n* = 209)	Rural (*n* = 200)	(11–12) (*n* = 172)	(13–14) (*n* = 237)	≤1^o^ (*n* = 149)	≥2^o^ (*n* = 260)
Q1. Care to your teeth? How?									
Tooth brushing	91.4	90.7	92.1	95.7	87.0 ^*∗∗*^	93.6	89.9	85.2	95.0 ^*∗∗*^
Rinsing with water	10.3	10.4	10.2	3.3	17.5 ^*∗∗∗*^	9.9	10.5	18.1	5.8 ^*∗∗∗*^
Using finger	9.8	10.4	9.3	2.4	17.5 ^*∗∗∗*^	18.1	11.0	19.5	4.2 ^*∗∗∗*^
Not brushing	1.0	1.0	9.0	1.0	1.0	1.7	0.4	0.7	1.2

Q2. Tooth cleaning devices?									
Plastic toothbrush	91.9	91.2	92.6	96.7	87.0 ^*∗∗∗*^	94.2	90.3	86.6	95.0 ^*∗∗*^
Chewing stick both plastic and chew stick	24.9	24.9	25.0	1.9	49.0 ^*∗∗∗*^	16.3	31.2 ^*∗∗*^	46.3	12.7 ^*∗∗∗*^
Cleaning with a finger	9.0	9.3	8.8	2.4	16.0 ^*∗∗∗*^	8.1	9.7	15.4	5.4 ^*∗∗*^
Just rinsing	14.2	11.9	16.2	2.4	26.5 ^*∗∗∗*^	11.0	16.5	29.5	5.4 ^*∗∗∗*^

Q3. Time to do tooth brushing?									
Before breakfast	91.9	90.2	93.5	90.0	94.0	87.2	95.4 ^*∗∗*^	94.0	90.8
Meal-time	18.6	18.1	19.0	7.7	30.0 ^*∗∗∗*^	15.7	20.7	32.9	10.4 ^*∗∗∗*^
When going to bed	24.9	27.5	22.7	17.2	33.0 ^*∗∗∗*^	20.9	27.8	33.6	20.0 ^*∗∗*^

Q4. Tooth cleaning frequency?									
Once/day	21.5	23.8	19.4	23.4	19.5	22.1	21.1	19.5	22.7
Twice/day	27.9	28.0	27.8	29.2	26.5	30.8	25.7	28.2	27.7
Three times/day	48.7	45.6	51.4	46.4	51.0	45.3	51.1	49.0	48.5
Once a week	2.0	2.1	1.9	1.4	2.5	2.3	1.7	2.7	1.5

Q5. Use of dentifrices-type?									
Toothpaste	78.2	77.2	79.2	91.9	64.0 ^*∗∗∗*^	85.5	73.0	62.4	87.3 ^*∗∗∗*^
Salt	16.4	16.7	316.7	8.1	25.0 ^*∗∗∗*^	16.9	16.0	24.2	11.9 ^*∗∗*^
Ashes	21.0	21.2	20.8	3.3	39.5 ^*∗∗∗*^	18.6	22.8	36.2	12.3 ^*∗∗∗*^
Sand	8.3	8.3	8.3	1.9	15.0 ^*∗∗∗*^	5.8	10.1	15.4	4.2 ^*∗∗∗*^

Q6. Tongue cleaning? (yes)	70.2	66.3	73.6	84.7	55.0 ^*∗∗∗*^	77.9	64.6 ^*∗∗*^	55.0	78.8 ^*∗∗∗*^

≤1°: primary school education and below; ≥2°: secondary school education and above. Statistically significant differences between the categories:  ^*∗∗∗*^ = *P* < 0.001,  ^*∗∗*^ = *P* < 0.01, and  ^*∗*^ = *P* < 0.05. No asterisk: statistically not significant (NS).

**Table 3:** Distribution of study participants in relation to knowledge about causes, symptoms, and prevention of dental caries and gingival disease by sociodemographic characteristics in percentages.

Oral condition	Age group (years)		Gender		Residence		Standard		Parents' education ^#^	
	11–12 years (*n* = 172)	13–14 years (*n* = 237)	Male (*n* = 193)	Female (*n* = 216)	Urban (*n* = 209)	Rural (*n* = 200)	Lower (*n* = 127)	Upper (*n* = 282)	≤1° (*n* = 149)	≥2° (*n* = 260)
Dental caries										
Causes	92.4	92.0	93.8	90.7	94.3	90.0	89.8	93.3	87.2	95.0 ^*∗∗∗*^
Symptoms	65.1	77.6 ^*∗∗*^	72.5	72.2	64.1	81.0 ^*∗∗∗*^	76.4	70.6	83.2	66.2 ^*∗∗∗*^
Prevention	84.9	86.1	88.6	82.9	89.0	82.0 ^*∗*^	80.3	87.9 ^*∗*^	79.2	89.2 ^*∗∗*^

Gingival disease										
Causes	90.7	94.1	92.7	92.6	89.8	94.0	91.9	93.5	94.6	91.5
Symptoms	83.7	86.5	86.5	84.3	84.2	86.5	86.6	84.8	86.6	84.6
Prevention	94.2	90.3	93.2	90.7	95.2	88.5 ^*∗*^	90.6	92.6	89.9	93.1

≤1°: primary school education and below; ≥2°: secondary school education and above. Statistically significant differences between the categories:  ^*∗∗∗*^ = *P* < 0.001,  ^*∗∗*^ = *P* < 0.01, and  ^*∗*^ = *P* < 0.05. No asterisk: statistically not significant (NS).

**Table 4:** Distribution of study participants in relation to toothache experience, disturbances in class, gingival bleeding, and swelling by sociodemographic characteristics.

Oral condition and disturbance in class	Age group (years)		Gender		Residence		Standard		Parents' education ^#^	
	11–12 years (*n* = 172)	13–14 years (*n* = 237)	Male (*n* = 193)	Female (*n* = 216)	Urban (*n* = 209)	Rural (*n* = 200)	Lower (*n* = 127)	Upper (*n* = 282)	≤1° (*n* = 149)	≥2° (*n* = 260)
Toothache	37.2	26.2 ^*∗*^	30.1	31.5	40.2	21.0 ^*∗∗∗*^	28.7	35.4	23.5	35.0 ^*∗*^
Gingival bleeding	25.6	23.6	27.5	21.8	30.1	18.5 ^*∗*^	22.8	25.2	22.1	25.8
Gingival swelling	25.6	19.0	20.7	22.7	27.3	16.0 ^*∗∗*^	19.7	22.7	18.8	23.5
Disturbed in class	20.9	17.3	16.6	20.8	19.6	18.0	19.7	18.4	20.1	18.1

Statistically significant differences between the categories:  ^*∗∗∗*^ = *P* < 0.001,  ^*∗∗*^ = *P* < 0.01, and  ^*∗*^ = *P* < 0.05. No asterisk: statistically not significant (NS).

**Table 5:** Binary logistic regression analyses of toothache and gingival bleeding in relation to knowledge of oral hygiene, oral hygiene practices, and demographic factors among primary schoolchildren in Lilongwe, Malawi.

Demographic and predisposing factors	Toothache				Gingival bleeding on tooth brushing			
	B	SE	Odds ratio	95% CI	B	SE	Odds ratio	95% CI
Not using plastic toothbrush	0.995	0.402	2.624	1.194–5.767^c^	1.222	0.403	3.395	1.540–7.484^b^
Not brushing before breakfast	0.706	0.384	2.027	0.955–4.301	0.772	0.388	2.165	1.012–4.632^c^
Residence: urban versus rural	1.032	0.237	2.806	1.765–4.461^a^	0.785	0.253	2.192	1.334–3.600^b^

B = beta weights (regression coefficient); SE: standard error; CI: confidence interval. Statistical significance: ^a^*P* < 0.001, ^b^*P* < 0.01, and ^c^*P* < 0.05. No asterisk: statistically not significant (NS).

## Data Availability

Quantitative data were used. Data used to support the findings of this study are available from the corresponding author upon request.
